# Supplementary material for: Synthesis, Characterization, and Study of the Antimicrobial Potential of Dimeric Peptides Derived from the C-Terminal Region of Lys49 Phospholipase A2 Homologs
Source: Toxins (Basel). 2024 Jul 5;16(7):308. doi: 10.3390/toxins16070308 (PMC11281518; doi:10.3390/toxins16070308)
Supplement: Supplementary file 1 [file toxins-16-00308-s001.zip › toxins-3048748-supplementary.pdf]

Synthesis, Characterization, and Study of the Antimicrobial Potential of Dimeric Peptides Derived from the C-Terminal Region of Lys^{49} Phospholipase A$_2$ Homologs

Gabriel F. H. Bicho, Letícia O. C. Nunes, Louise Oliveira Fiametti, Marcela N. Argentin, Vitória T. Candido, Ilana L. B. C. Camargo, Eduardo M. Cilli and Norival A. Santos-Filho

Table S1: Method used in peptide synthesis.

Peptide	Sequence	Resin	Cleavage cocktail	Peptide purification method
(p-MtII)$_2$K	(KKYRYYLKPL)$_2$K	Rink amide MBHA	95% TFA, 2,5% H_2O, 2,5% TIS	5-60% in 120 minutes
(p-EM-2)$_2$K	(KKWRWWLKAL)$_2$K	Rink amide MBHA	95% TFA, 2,5% H_2O, 2,5% TIS	20-60% in 120 minutos
* (p-ACL)$_2$K	(KKYKAYFKFK)$_2$K	Rink amide MBHA	95% TFA, 2,5% H_2O, 2,5% TIS	10-60% in 120 minutos
(p-AppK)$_2$K	(KKYKAYFKLK)$_2$K	Rink amide MBHA	95% TFA, 2,5% H_2O, 2,5% TIS	15-45% in 120 minutos

Figure S1: Chromatogram of synthesized and purified peptides determined by HPLC.

a) (p-MtII)$_2$K.

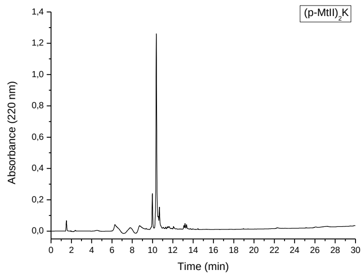

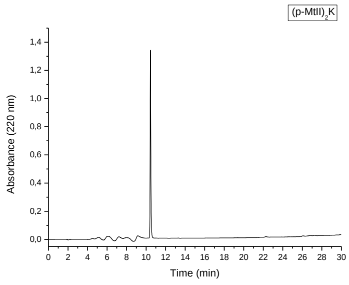

b) (p-EM-2)$_2$k.

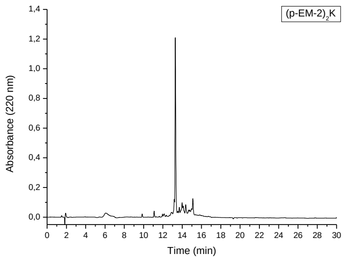

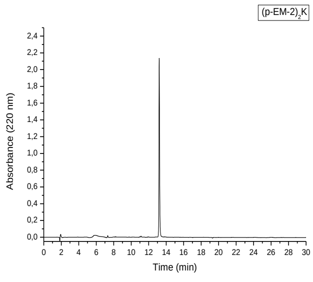

c) (ACL)$_2$k.

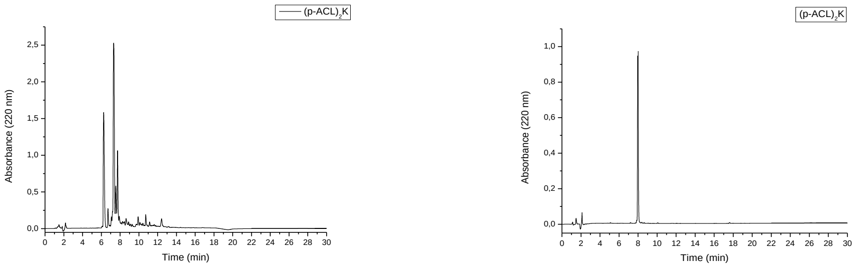

d) (AppK)$_2$k.

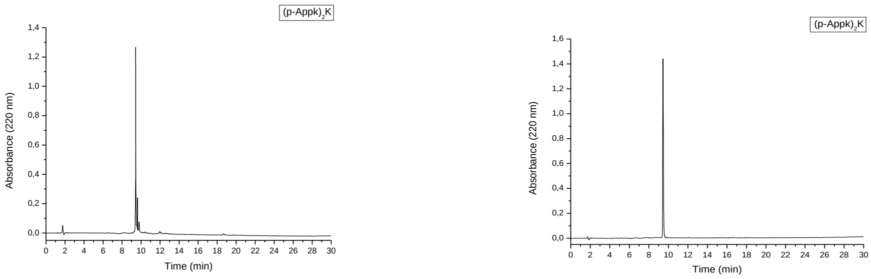

Figure S2: Mass spectra of pure peptides.

a) (p-MtII)$_2$K

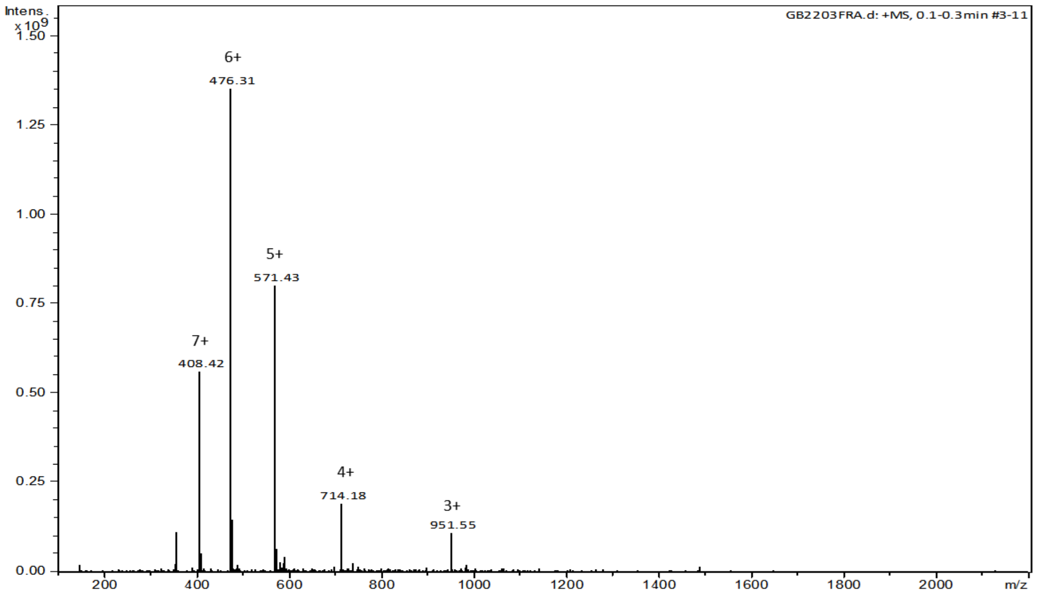

b) (p-EM-2)$_2$K

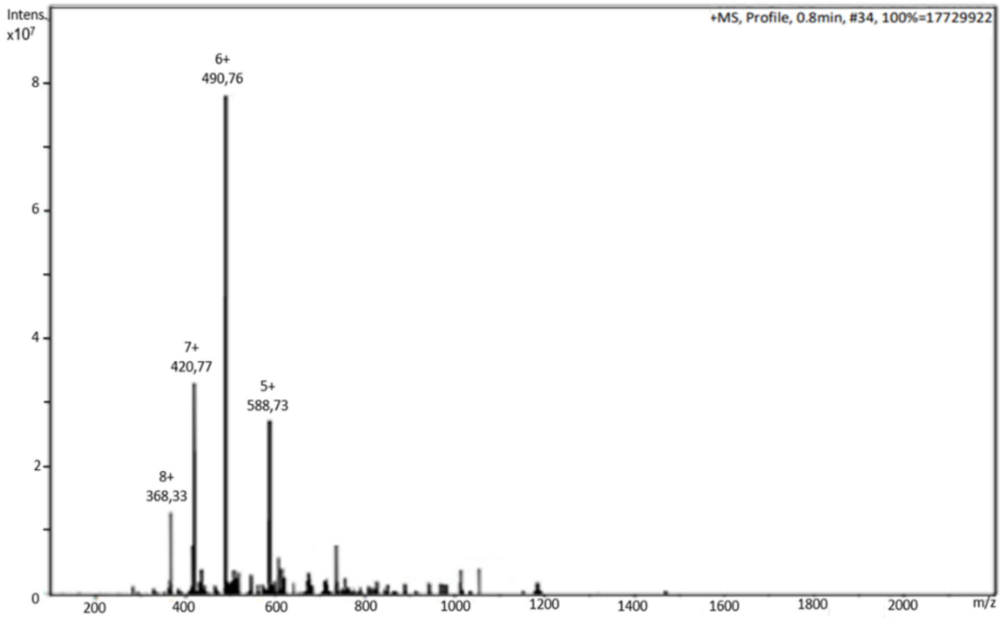

c) (p-ACL)$_2$K

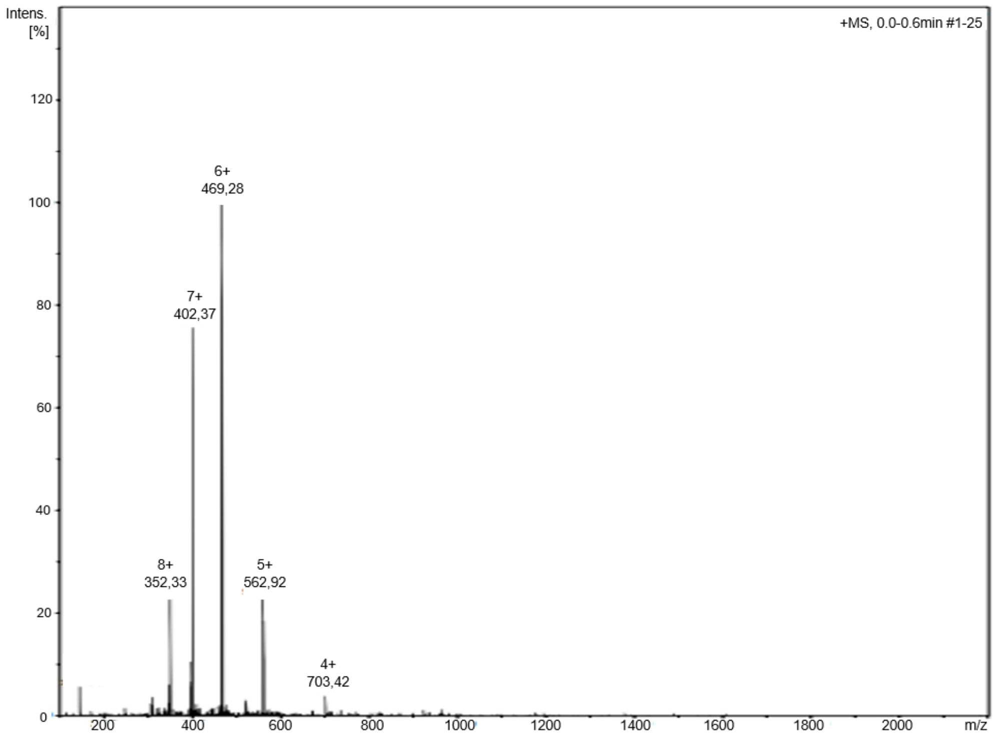

d) (p-AppK)$_2$K

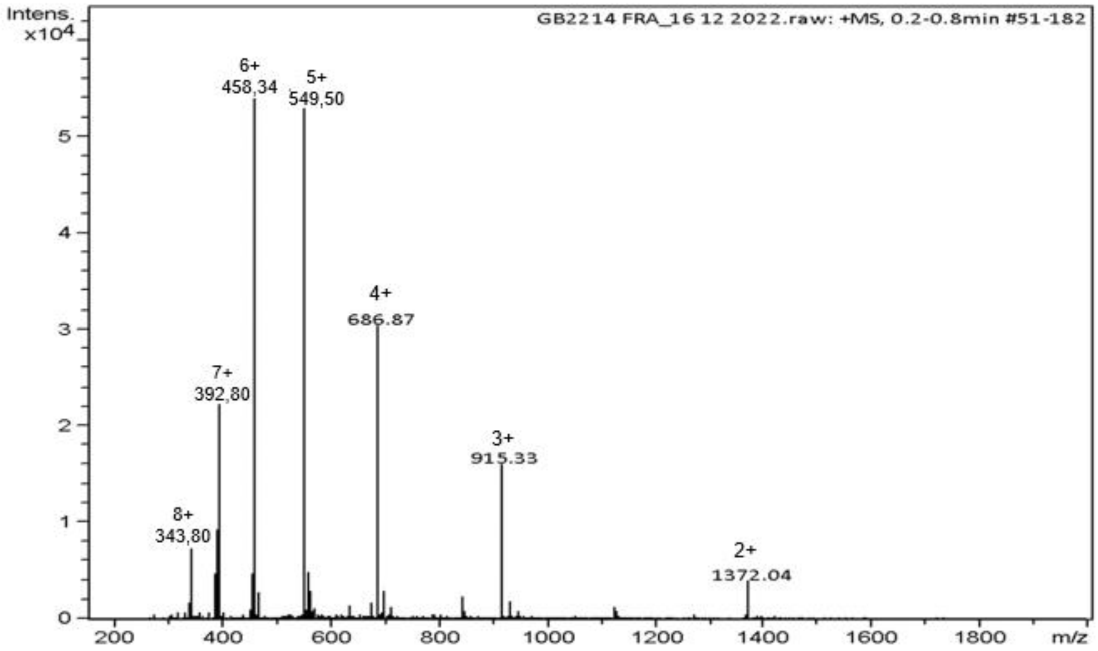

Intens.
x10⁴
GB2214 FRA_16 12 2022.raw: +MS, 0.2-0.8min #51-182
6+
458,34
5+
549,50
4+
686.87
7+
392,80
3+
915.33
8+
343,80
2+
1372.04
0
1
2
3
4
5
200
400
600
800
1000
1200
1400
1600
1800
m/z

Table S2. Susceptibility profile of the Gram-positive strains tested in this study using antibiotics currently available in the market

Antibiotics	*S. epidermidis* ATCC 35984		*S. aureus* ATCC 25923		*S. aureus* ATCC 8095		*E. faecalis* ATCC 29212		*E. faecium* ATCC 700221	
	MIC	interpretation	MIC	interpretation	MIC	interpretation	MIC	interpretation	MIC	interpretation
Ampicillin	>4	R	N.R.	N.R.	N.R.	N.R.	≤2	S	>8	R
Cefoxitin	≤0,5	R	≤1	S	2	S	N.R.	N.R.	N.R.	N.R.
Ceftaroline	N.R.	N.R.	≤0,5	S	≤0,5	S	N.R.	N.R.	N.R.	N.R.
Ciprofloxacin	≤0,5	I	≤0,5	S	≤0,5	S	1	S	N.R.	N.R.
Clindamycin	>2	R	≤0,5	S	≤0,5	S	N.R.	N.R.	N.R.	N.R.
Chloramphenicol	N.R.	N.R.	8	S	8	S	N.R.	N.R.	N.R.	N.R.
Daptomycin	≤1	S	≤1	S	≤1	S	N.R.	N.R.S	N.R.	N.R.
Erythromycin	>4	R	≤0,25	S	≤0,25	S	N.R.	N.R.	N.R.	N.R.
Streptomycin-Sin	N.R.	N.R.	N.R.	N.R.	N.R.	N.R.	>1000	R	>1000	R
Gentamicin	>8	R	≤2	S	≤2	S	N.R.	N.R.	N.R.	N.R.
Gentamicin-Sin	N.R.	N.R.	N.R.	N.R.	N.R.	N.R.	≤500	S	>500	R
Linezolid	2	S	≤1	S	≤1	S	2	S	2	S
Minocycline	≤1	S	≤1	S	≤1	S	N.R.	N.R.	N.R.	N.R.
Nitrofurantoin	N.R.	N.R.	N.R.	N.R.	N.R.	N.R.	≤16	S	N.R.	N.R.
Oxacillin	>2	R	≤0,25	S	0,5	S	N.R.	N.R.	N.R.	N.R.
Penicillin G	>1	R	≤0,125	S	≤0,125	S	N.R.	N.R.	N.R.	N.R.
Teicoplanin	≤2	S	≤1	S	≤1	S	≤1	S	>16	R
Trimethoprim-Sulfamethoxazole	>2/38	R	≤0,5/9,5	S	≤0,5/9,5	S	N.R.	N.R.	N.R.	N.R.
Vancomycin	1	S	1	S	1	S	2	S	>16	R

*N.R. – not reported, either because the MIC is reported only with no interpretation, or the test is not reported because there is insufficient information to have a breakpoint; S, Susceptible; I, Susceptible increased exposure; R, resistant.

Table S3. Susceptibility profile of the Gram-negative strains tested in this study using antibiotics currently available in the market

Antibiotics	*K. pneumoniae* ATCC 700603		*E. coli* ATCC 25922		*A. baumannii* ATCC 19606		*P. aeruginosa* ATCC 27853	
	MIC	interpretation	MIC	interpretation	MIC	interpretation	MIC	interpretation
Amikacin	≤4	S	≤4	S	>16	R	≤8	S
Ampicillin-Sulbactam	16/8	I	≤4/2	S	N.R.	N.R.	N.R.	N.R.
Cefepime	2	S	≤1	S	N.R.	N.R.	2	≤1
Ceftazidime	>8	R	≤1	S	N.R.	N.R.	4	I
Ceftazidime-Avibactam	1/4	S	≤0,25/4	S	N.R.	N.R.	N.R.	N.R.
Ceftolozano-Tazobactam	≤1/4	S	≤1/4	S	4/4	N.R.	N.R.	N.R.
Ceftriaxone	4	R	≤1	S	N.R.	N.R.	N.R.	N.R
Cefuroxime	>16	R	≤4	S	N.R.	N.R.	N.R.	N.R.
Ciprofloxacin	0,5	I	≤0,25	S	1	I	0,5	I
Colistin	≤1	S	≤1	S	≤1	S	≤1	S
Ertapenem	≤0,25	S	≤0,25	S	N.R.	N.R.	N.R.	N.R.
Gentamicin	>4	R	≤1	S	>4	R	N.R.	N.R.
Imipenem	≤0,25	S	≤0,25	S	8	R	4	I
Levofloxacin	1	I	≤0,5	S	≤0,5	S	≤1	I
Meropenem	≤0,5	S	≤0,5	S	1	S	≤0,5	S
Piperacillin-Tazobactam	16/4	S	≤4/4	S	N.R.	N.R.	≤4/4	I
Trimethoprim-Sulfamethoxazole	≤1/19	S	≤1/19	S	>4/76	R	N.R.	N.R.

*N.R. – not reported, either because the MIC is reported only with no interpretation, or the test is not reported because there is insufficient information to have a breakpoint; S, Susceptible; I, Susceptible increased exposure; R, resistant.
